# Necrotizing Soft Tissue Infections, the Challenge Remains

**DOI:** 10.3389/fsurg.2021.721214

**Published:** 2021-09-09

**Authors:** Femke Nawijn, Falco Hietbrink, Andrew B. Peitzman, Luke P. H. Leenen

**Affiliations:** ^1^Department of Surgery, University Medical Center Utrecht, Utrecht, Netherlands; ^2^Department of Surgery, University of Pittsburgh, Pittsburgh, PA, United States

**Keywords:** mortality, necrotizing soft tissue infections, necrotizing fasciitis, outcomes, presentation NSTIs

## Abstract

**Background:** Necrotizing Soft Tissue Infections (NSTIs) are uncommon rapidly spreading infection of the soft tissues for which prompt surgical treatment is vital for survival. Currently, even with sufficient awareness and facilities available, ambiguous symptoms frequently result in treatment delay.

**Objectives:** To illustrate the heterogeneity in presentation of NSTIs and the pitfalls entailing from this heterogeneity.

**Discussion:** NSTI symptoms appear on a spectrum with on one side the typical critically ill patient with fast onset and progression of symptoms combined with severe systemic toxicity resulting in severe physical derangement and sepsis. In these cases, the suspicion of a NSTI rises quickly. On the other far side of the spectrum is the less evident type of presentation of the patient with gradual but slow progression of non-specific symptoms over the past couple of days without clear signs of sepsis initially. This side of the spectrum is under represented in current literature and some physicians involved in the care for NSTI patients are still unaware of this heterogeneity in presentation.

**Conclusion:** The presentation of a critically ill patient with evident pain out of proportion, erythema, necrotic skin and bullae is the classical presentation of NSTIs. On the other hand, non-specific symptoms without systemic toxicity at presentation frequently result in a battery of diagnostics tests and imaging before the treatment strategy is determined. This may result in a delay in presentation, delay in diagnosis and delay in definitive treatment. This failure to perform an adequate exploration expeditiously can result in a preventable mortality.

## Introduction

Necrotizing soft tissue infections (NSTIs) are an ancient challenge, considering that Hippocrates first described the infection in the 5^th^ century BC. He reported the following clinical observation “many even while undergoing treatment suffered from severe inflammation, and the erysipelas would quickly spread widely in all directions. Flesh, sinews and bones fell away in large quantities … there were many dead” (1). The disease Hippocrates described as “erysipelas all over the body” later became known by a broad range of names, such as “the flesh-eating infection,” necrotizing fasciitis, Fournier gangrene and severe necrotizing soft tissue disease (SNSTD) (2–4). All of those terms for this uncommon, rapidly spreading, progressive and potentially lethal infection of the soft tissues are currently represented in the internationally accepted term necrotizing soft tissue infection (NSTI) (5, 6). The incidence of NSTIs in the United States is estimated to be 4.0 to 10.3 per 100,000 person years, while the incidence of the Dutch population, probably comparable to most European countries, is 1.1–1.4 per 100,000 person years (7, 8). Unfortunately, the statement of Hippocrates that many died, remains current. Even though the mortality rates were decreased by half over the past decades, the last two decades the mortality rate for NSTIs remained unchanged, at ~20% (9, 10). Two recent meta-analyses reported that prompt surgical treatment is vital for further reduction of mortality (9, 11). However, even with sufficient awareness and facilities available, ambiguous symptoms may still result in delay, perhaps even more so when compared to a patient presenting critically ill (12, 13). In fact, NSTI are misdiagnosed initially more often than not due to pitfalls such as absence of fever, absence of cutaneous manifestations (Tables 1, 2), intact skin in the area (no skin defect as entry), attributing pain to an injury or procedure, non-diagnostic/non-specific imaging results, or ascribing the systemic signs to other causes (6, 38). Treatment delay is often preceded by a patient and/or diagnostic delay, which is thought to be even greater in case of first presentation with ambiguous symptoms (38). As Hippocrates described, “Fever was sometimes present and sometimes absent”; patients with NSTI present with variable signs and symptoms, and only a limited number of patients present with the classic symptoms (1, 4, 5).

**Table 1:** Overview of large necrotizing soft tissue infection studies published since 2000 reporting on microbiology, time to surgery and/or presenting symptoms.

									**Symptoms upon presentation**								
**References**	**Country**	**Included patients **	**Time to surgery (in hours) **	**Age (in years) **	**Polymicrobial infections (type I) **	**Monomicrobial NSTIs (type II) **	**No cultures **	**Mortality **	**Tachycardia **	**Hypotension **	**Fever **	**Swelling **	**Erythema **	**Pain **	**Skin necrosis **	**Bullae **	**crepitus **
**Bair** (14)	Taiwan	106	64 ± 58	58 ± 14	57%	16%	20%	**17%**	-	-	-	-	-	-	-	-	-
**Chen** (15)	Taiwan	323	-	58 ± 15	55%	45%	14%	**16%**	47%	13%	32%	-	78%	81%		41%	-
**Chen** (16)	Canada	60	16	54 ± 18	33%	42%	25%	**22%**	-	-	-	92%	87%	-	-	28%	-
**Dworkin** (17)	USA	80	-	-	39%	30%	25%	**15%**	31%	22%	43%	78%	75%	-	24%	33%	14%
**Frazee** (18)	USA	122	8^a^	44 ± 11	47%	38%	15%	**16%**	59%	21%	44%	-	80%	54%	24%	12%	7%
**Hong** (19)	Taiwan	195	5 (IQR 2 – 14)	55 ± 16	-	-	-	**14%**	52%	-	25%	-	95%	98%	-	55%	-
**Hua** (20)	France	106	-	62 (IQR 49 – 75)	63%	30%	7%	**28%**	-	-	-	98%	96%	-	75%	58%	21%
**George** (21)	USA	78	22 ± 21	49 ± 20	36%	42%	16%	**10%**	-	-	-	-	-	-	-	-	-
**Huang** (22)	Taiwan	472	-	60 ± 14	34%	57%	9%	**12%**	-	-	40%	84%	61%	74%	16%	13%	5%
**Jabbour** (23)	Qatar	331	-	51 ± 15	27%	44%	23%	**26%**	-	-	67%	78%	-	68%	-	-	-
**Keeley** (24)	USA	138	-	46^a^	42%	58%	0%	**15%**	-	-	-	-	-	-	8%	12%	13%
**Kha** (25)	NC	67	-	54^b^	39%	46%	15%	**24%**	-	-	40%	78%	72%	76%	9%	39%	6%
**Kiralj** (26)	Serbia	216	-	52^b^	80%	20%	-	**14%**	-	-	-	85%	56%	85%	14%	76%	2%
**Kongkaewpaisan** (27)	USA	91	5 (IQR 3 – 9)	53^b^	-	-	-	**10%**	81%	-	28%	-	90%	94%	-	14%	12%
**Krieg** (28)	Germany	64	-	54^b^	52%	37%	11%	**33%**	69%	48%	-	77%	80%	-	36%	16%	17%
**Kulasegaran** (29)	NZ	138	10^a^	-	50%	50%	0%	**22%**	-	-	-	-	-	-	-	-	-
**Latifi** (30)	USA	115	27^b^	55 ± 18	-	-	-	**17%**	43%	41%	49%	45%	80%	72%	63%	5%	7%
**Misiakos** (31)	Greece	62	13^b^	64^b^	-	-	-	**18%**	34%	15%	31%	74%	69%	90%	47%	23%	10%
**Okoye** (32)	USA	64	35 ± 6	49± 13	61%	33%	6%	**14%**	-	-	-	-	-	-	-	-	-
**Salvador** (33)	Philippines	67	-	-	34%	36%	30%	**36%**	-	8%	63%	76%		94%	33%	35%	14%
**Van Stigt** (34)	NL	123	11 ± 13	58 ± 14	42%	56%	2%	**32%**	-	-	-	86%	82%	69%	-	20%	13%
**Wang** (35)	Taiwan	115	-	54 (IQR 44−75	17%	61%	22%	**21%**	-	-	76%	-	80%	73%	-	22%	6%
**Wong** (36)	Singapore	89	-	56^b^	54%	28%	18%	**21%**	74%	18%	53%	-	100%	98%	14%	45%	14%
**Pooled results**		3222	20	55	45%	43%	12%	**18%**	53%	21%	45%	80%	76%	78%	26%	31%	9%

a *median;*

b *mean.*

*IQR = Interquartile range; NC = New Caledonia; NL = The Netherlands; NZ = New-Zealand; USA = United States of America.Studies were eligible for inclusion in this table if they included over 50 patients and two out of three items were reported: (1) time to surgery, (2) percentage of poly- and monomicrobial infections and (3) symptoms upon presentation. Studies were identified using the articles previously screened for our own meta-analysis (9) and was updated using the same search strategy, furthermore studies were identified from two previous systematic reviews on NSTIs*.

**Table 2:** Summary of occurrence of cutaneous or systemic symptoms of necrotizing soft tissue infections (14–37).

**Cutaneous symptoms**	Soft tissue edema (“woody induration”)	80%
	Erythema	76%
	Severe pain or tenderness - out of proportion to signs - crescendo pain	78%
	Bullae	31%
	Skin necrosis	26%
	Crepitus	9%
**Systemic symptoms**	Tachycardia	53%
	Fever	45%
	Hypotension	21%

## Clinical Perspective

To illustrate the heterogeneity in presentation, three patients from a database previously published by our study group are presented, and a review of current literature is presented (Table 1) (39).

**First**, a 69-year-old female patient with rheumatoid arthritis, presented with a painful and swollen leg since that morning. In the evening, she started vomiting and presented to the hospital with swelling, erythema and blue-purple discoloration of the entire lower leg and two large bullae, was hypotensive (mean arterial pressure of 59 mmHg), had tachycardia, elevated lactate levels (2.9 mmol/L) and base deficit of 7 mmol/L. Within 6 h of first onset of symptoms, she was taken to the operating room and diagnosed with a NSTI caused by group A streptococcus (GAS).

**Second**, 53-year-old male patients with chronic obstructive pulmonary disease presented with acute and progressive dyspnea for 2 days. As the patient's history was obtained, it was discovered that he had a swollen leg for 4 days, which the general practitioner previously diagnosed as a bursitis. Upon presentation, the patient already had severe tachypnea and tachycardia, was his entire leg swollen, pale and warm, and had a severe metabolic acidosis (base deficit −19 mmol/L, lactate 13.7 mmol/L). At the emergency department, he further deteriorated and was intubated. Due to uncertainty about the focus of the infection, a computed tomography (CT) scan was obtained which showed gas formation in the soft tissues of the upper leg. Consequently, the patient was taken to the operating room and a NSTI caused by multiple anaerobic and aerobic micro-organism was diagnosed.

**Third**, 68-year-old male patients with diabetes mellitus, hypertension and a previous kidney transplant presented with a swollen upper leg with localized erythema without tachycardia, tachypnea or hypotension. The patient reported having malaise since last week and to have erythema of the leg for 2 days. The symptoms were diagnosed as cellulitis and the patient was admitted. Over the next 24 h the erythema and pain gradually increased, the patient developed dyspnea and tachycardia and developed a metabolic acidosis (base deficit 6.9 mmol/L, 3.9 mmol/L at presentation). The patient was eventually taken to the operating room and a NSTI caused by an aerobic micro-organism was diagnosed.

## Discussion

### The Outer Ranges of the NSTI Presentation Spectrum

As described above, two sides of the NSTI presentation spectrum can be seen. On one side, the typical critically ill patient with fast onset and progression of symptoms (e.g., pain out of proportion, erythema, bullae) combined with severe systemic toxicity resulting in systemic inflammatory response syndrome (SIRS) and sepsis (patient 1). In these cases, the suspicion of a NSTI rises quickly due to its typical and notorious presentation. The response by care providers is all hands-on deck since the consequences, mortality and amputations, are well-known. The sepsis protocol is immediately initiated and the patient is rapidly transported to the operating room without additional testing or imaging at the emergency department, since delay to the first debridement increases the need for subsequent debridements, as well as increases the risk at mortality (9, 30, 40, 41). On the other far side of the spectrum is the patient with gradual but slow progression of non-specific symptoms over the past couple of days without evident signs of sepsis initially. However, when these patients finally present, they can either deteriorate rapidly (patient 2) or may continue to deteriorate slowly (patient 3). This side of the spectrum is under represented in current literature and some physicians involved in the care for NSTI patients are still unaware of this heterogeneity in presentation. Both patient subtypes have the same severe infection, and both require immediate (surgical) treatment, but the challenge remains to treat them equally efficient.

### Causes of Variation in Presentation

The precise cause of this misleading contrast in presentation remains unclear. Recent literature described differences in patient demographics between the different micro-organisms isolated from NSTIs (17, 36, 39). This strengthens the hypothesis that the causative micro-organisms influences the different types of presentation, since the variance in early or late systemic toxicity was described to depend on the strain of bacteria and toxins produced (2). Causative micro-organisms of NSTIs are generally categorized in three categories, based on the number of different micro-organisms found and less so the determination of the micro-organisms. Type I is polymicrobial and generally consists of various species of gram-positive cocci, gram-negative rods and anaerobes (2, 10). Type II is monomicrobial with GAS being the most common microbe found (39, 42). Recently an attempt was made to categorize specific strains of bacteria in their presentation, as Type III consists of more rare isolated microbes. Most commonly, this involves infections with the *Vibrio vulnificus or Clostridium* species. However, most authors still categorize them as type I due to it either being a gram-negative or anaerobic bacteria (2, 10, 36). The frequency of presentation of each of these types differ across geographical areas and populations, whereas *Vibrio* NSTIs are more often seen in Asia, GAS NSTIs more often in Europe and a relatively high frequency of methicillin-resistant *Staphylococcus aureus* NSTIs are seen in the United States (39, 43, 44).

Systemic toxicity of polymicrobial infections is most commonly dependent on the mechanism of microbial synergy and not necessarily on toxins. This process requires time and does not immediately cause a fulminant presentation of NSTIs as mainly seen in type II NSTIs. Micro-organisms causing monomicrobial infections do not require synergy. They frequently are able to produce (multiple) toxins on their own, causing rapid systemic toxicity (45). Generally, physicians associate NSTIs with early systemic toxicity due to its rapidly progressive and destructive character. However, this is mostly exemplary for NSTI based on GAS, *Clostridium* spp. and *Vibrio* spp., while in most polymicrobial NSTIs signs of systemic toxicity commonly occur late or not at all (2, 46). Compared to type I NSTIs, patients with type II NSTIs tend to be younger, healthier and more commonly have a history of trauma, surgery or IV drug use as causative event (2, 39). Patients with type I NSTIs usually have more and severe comorbidities (such as diabetes mellitus, peripheral vascular disease, chronic renal failure) (2, 6, 39). This could indicate that especially younger and healthier patients present with early systemic toxicity, while notable the older patients with severe and/or multiple comorbidities present with late systemic toxicity and have a higher risk of expiring (3, 28, 39, 42). Therefore, the absence of systemic toxicity can be misleading, but should be kept in mind as diagnostic pitfall.

### Types of Delay Due to Variation in Presentation

#### Patient Delay

Patients without signs of sepsis and indifferent symptoms will less urgently seek medical care, causing patient delay and therefore treatment delay. On the other hand, patients with high fever, low blood pressure and high heart rates are likely to rush to the emergency department.

#### Diagnostic Delay

Furthermore, when critically ill patients present, they will receive priority for diagnostic tests and treatment. This is seen in a study comparing GAS NSTIs with non-GAS NSTIs. In the study, 77% of the GAS NSTI patients had surgical exploration within 24h compared to 66% of the non-NSTI patients (39). This suggests that GAS NSTIs, or type II NSTIs, are diagnosed earlier and therefore treated faster. It can be considered common practice that the critically ill patients are rapidly seen by (senior) consults, while the more stable patients are commonly first seen by the (less experienced) residents. Another source of delay, caused by a non-specific presentation of a NSTI, is the physician does not recognize the severity of the presentation and first starts with a myriad of diagnostic tests and in some cases imaging. The use of imaging for diagnosis of NSTIs is controversial. Some studies advocate the use of imaging modalities, such as plain radiographs or CT scan. A recent meta-analysis showed limited added value of imaging. In addition, imaging can significantly delay treatment due to waiting times for the scan itself and due to the time required for interpretation of the scans by a radiologist (47). However, this can take up to several hours, which could be a significant proportion of the recommended maximum time from presentation to surgery for NSTIs (9, 48, 49). One of the few situations in which it can be contemplated to order a CT scan, is when an abdominal source (e.g., gastro-intestinal fistulas to the abdominal wall) of the NSTI is suspected, since this would require intra-peritoneal source control (50). Nonetheless, logistics for an emergency CT-scan should be optimal since imaging should not delay surgical consultation or intervention (5). Delay to first debridement or inadequate first debridement increases mortality (9, 11).

#### Treatment Delay

Besides diagnostic delay, treatment delay can occur in case of ambiguous presentation without evident signs of sepsis, when patients are first seen by non-surgical specialties, causing tendency toward a non-surgical course of treatment. A recent review showed that 71.4% of the NSTIs are misdiagnosed on initial evaluation (38). This misdiagnosis frequently results in a wait-and-see course of treatment with intravenous antibiotics, without surgical involvement for the vital source control (4, 5).

## Surgical Consequences of Variation in Presentation

When the decision is made to transport the patients to the operating room, there are still certain pitfalls to avoid during surgical exploration. First, skin, subcutaneous tissue, fascia and the muscle deep to the fascia must all be examined. In 57% of the NSTI cases, findings such as gray necrotic tissue, dishwater pus, and lack of bleeding or tissue resistance will be seen upon first evaluation of the soft tissues, which evidently confirms the diagnosis NSTI (3). However, in the other 43%, only ambiguous findings are seen upon macroscopic evaluation. In these cases, the algorithm of triple diagnostics might be of added value. This algorithm indicates that in case of non-diagnostic macroscopic findings, a Gram stain and a fresh frozen section of a full-thickness incisional biopsy are intra-operatively assessed (10, 51–54). However, in case of a less strong indication for surgical exploration, based on an indifferent presentation and therefore only moderate suspicion for NSTI, the surgeon might be tempted to perform a less invasive exploration with a smaller and/or more superficial incision. This will result in only partial evaluation of the tissue layers and increases the odds that, based on this (limited) macroscopic evaluation, it is wrongfully discarded as a non-necrotizing infection without additional (microscopic) testing such as histopathological or Gram stain assessment (51). While in case of a critically ill patients without evident macroscopic signs, it is more likely that the surgeon would want more reassurance intra-operatively.

Additionally, the skin sparing approach for debridement is increasing in popularity due to its reconstructive advantage. A recent cohort study showed no increase or reduction in mortality or in post-operative complications when skin spared debridement is performed. However, 85% of the patients in this study were transferred from outside hospitals indicating that these patients were stable enough to survive a significant delay caused by transfer (55). Surgeons must be aware that, although results seem promising, this approach should not result in delay in source control or resuscitation.

## Conclusion

NSTI have an heterogeneous presentation with a spectrum ranging from the classical presentation of a critically ill patient with evident pain out of proportion, erythema, necrotic skin and bullae, which should be recognized by all care providers. While on the other side of the spectrum are the, non-specific symptoms without systemic toxicity at presentation which frequently result in a battery of diagnostics tests and imaging before the treatment strategy is determined. This results in a delay in presentation (patient seeking medical aid) and delay in diagnosis and delay in definitive treatment. Confirmatory diagnosis of NTSI should occur in the operating room (not the radiology suite) with adequate exploration of all layers of areas of concern. Failure to perform an adequate exploration expeditiously can result in a preventable mortality.

## Data Availability Statement

The original contributions presented in the study are included in the article/supplementary material, further inquiries can be directed to the corresponding author/s.

## Ethics Statement

The studies involving human participants were reviewed and approved by METC Utrecht. Written informed consent for participation was not required for this study in accordance with the national legislation and the institutional requirements.

## Author Contributions

FN and FH came up with the article concept. FN, FH, LL, and AP collected the literature which FN and FH analyzed and incorporated the literature. FN and FH drafted the manuscript, which all authors critical revised.

## Conflict of Interest

The authors declare that the research was conducted in the absence of any commercial or financial relationships that could be construed as a potential conflict of interest.

## Publisher's Note

All claims expressed in this article are solely those of the authors and do not necessarily represent those of their affiliated organizations, or those of the publisher, the editors and the reviewers. Any product that may be evaluated in this article, or claim that may be made by its manufacturer, is not guaranteed or endorsed by the publisher.
